# The Use of Endoscopic Ultrasonography in Inflammatory Bowel Disease: A Review of the Literature

**DOI:** 10.3390/diagnostics13030568

**Published:** 2023-02-03

**Authors:** Răzvan-Cristian Statie, Dan Nicolae Florescu, Dan-Ionuț Gheonea, Bogdan Silviu Ungureanu, Sevastița Iordache, Ion Rogoveanu, Tudorel Ciurea

**Affiliations:** 1 Department of Gastroenterology, University of Medicine and Pharmacy of Craiova, 200349 Craiova, Romania; 2 Research Center of Gastroenterology and Hepatology, University of Medicine and Pharmacy of Craiova, 200638 Craiova, Romania

**Keywords:** ultrasonography, endoscopic ultrasound, inflammatory bowel disease, Crohn’s disease, ulcerative colitis

## Abstract

The diagnosis of inflammatory bowel disease (IBD) can sometimes be challenging. By corroborating clinical, imaging and histological data, the two main entities of IBD, ulcerative colitis and Crohn’s disease (CD), can be differentiated in most cases. However, there remains 10–20% of patients where the diagnosis cannot be accurately established, in which case the term “IBD unclassified” is used. The imaging techniques most used to evaluate patients with IBD include colonoscopy, ultrasonography and magnetic resonance imaging. Endoscopic ultrasonography is mainly recommended for the evaluation of perianal CD. Through this work, we aim to identify other uses of this method in the case of patients with IBD.

## 1. Introduction

The diagnosis of inflammatory bowel disease (IBD) can sometimes be challenging. By corroborating clinical, imaging and histological data, the two main entities of IBD, ulcerative colitis (UC) and Crohn’s disease (CD), can be differentiated in most cases. However, there remains 10–20% of patients where the diagnosis cannot be accurately established by conventional methods [1], in which case the term “IBD unclassified” (IBDU) is used [2].

Colonoscopy provides information about the macroscopic aspect of the colonic mucosa and allows us to take biopsies for histopathological confirmation of the diagnosis, still being the primary investigation for the identification and evaluation of patients with IBD [3]. In UC, the inflammatory process mandatory affects the rectum and can extend proximally, affecting the left colon and other segments of the large bowel; the characteristic lesions are continuous and involve the colonic mucosa circumferentially, and there is a clear demarcation between the parts affected by the disease and the normal ones; microscopically, the inflammatory process is limited to the mucosa, muscularis mucosae and submucosa [4]. CD can affect any segment of the digestive tract, but most frequently, the lesions are located in the terminal ileum and colon, while the rectum is spared in most cases; the characteristic lesions have a segmental distribution, being separated by areas of normal-looking mucosa; histologically, the inflammatory process is transmural and can affect all layers of the intestinal wall. Therefore, patients with CD may associate the development of fistulas and abscesses [4].

A disadvantage of colonoscopy is that it does not allow for complete transmural assessment of the intestinal wall, which is particularly important, especially in the case of patients with CD, so this role falls to other imaging investigations.

Cross-sectional imaging techniques recommended by the European Crohn’s and Colitis Organization (ECCO) for the evaluation of patients with IBD include intestinal ultrasound (IUS), magnetic resonance imaging (MRI), computed tomography (CT), endoanal ultrasonography (EAUS) and transperineal ultrasonography (PUS) [5]. IUS is increasingly used for monitoring patients with IBD, being widely available, relatively low-cost and a non-irradiating method, but it has certain limitations. It is an operator-dependent method, and the quality of the obtained ultrasonographic image can be influenced by intestinal gas, increased abdominal circumference and motion artefacts [6]. Endoscopic ultrasound (EUS) can overcome some of these barriers. This method has the advantage of placing the ultrasound transducer in direct contact with the intestinal wall [1].

An evaluation of the colon using EUS can be performed with the help of two types of echoendoscopes, radial and linear. These endoscopes are side-viewing instruments, which is why, classically, EUS of the lower digestive tract allows examination mainly of the rectum and anal canal [7]. For the evaluation of the anal canal, a radial echoendoscope is preferred, and for the rectal and pararectal regions, the linear echoendoscope is recommended [8]. Sometimes the use of the radial type is initially preferred to perform a complete examination, with the subsequent use of the linear echoendoscope to perform a therapeutic maneuver [8]. The ultrasonographic evaluation of the entire colon is currently possible with new-generation instruments, such as the forward-viewing linear echoendoscope [7]. Moreover, ultrasonographic examination during conventional colonoscopy is possible by using EUS mini probes, which can be inserted through the colonoscope’s working channel (instrument channel) [7]. Standard EUS systems use frequencies of 7.5 MHz and 12 MHz, while in the case of mini probes, the ultrasound frequency can reach up to 20 MHz [9] or even more. Some authors suggest that when the colonic lumen is large, the use of a conventional EUS system is preferred; however, in the case of a smaller diameter of the lumen, due to the higher resolution, for a better characterization of the layers of the intestinal wall, the use of mini probes is recommended [10], which are also particularly useful in the case of CD complicated with luminal stenoses.

During the EUS examination, the colorectal wall is typically made up of five concentric layers, being represented from inside to outside as follows: layer I—hyperechoic = mucosa; layer II—hypoechoic = muscularis mucosae; layer III—hyperechoic = submucosa; layer IV—hypoechoic = muscularis propria; and layer V—hyperechoic = serosa. The thickness of the colorectal wall in normal subjects can vary depending on the intestinal segment evaluated. Thus, the colon wall can have an average thickness of 1.40 mm. In contrast, the rectal wall can reach up to 2.14 mm, with significantly higher thickness of the mucosa, submucosa and muscularis propria in this segment [10].

According to current guidelines, the main recommendation for using EUS in IBD is evaluating perianal CD [11]. Therefore, this review aims to identify other uses of this method in the case of IBD patients.

## 2. Materials and Methods

To describe the role of EUS in IBD, we performed a search of available publications in the PubMed database, using the following combination of keywords: (“endoscopic ultrasound” or “endoscopic ultrasonography”) AND (“inflammatory bowel disease” or “Crohn’s disease” or “ulcerative colitis”). The search was not limited to a specific time interval in terms of the publication date of the articles. The exclusion criterion was represented by non-English language. A total of 66 articles were identified that could have provided valuable details for the studied topic of interest. Finally, 47 articles were selected for writing the review, from which the necessary information was extracted.

## 3. Perianal Crohn’s Disease

Approximately 30% of CD patients may develop the perianal disease during their lifetime [12]. This term refers to the presence of lesions of the anal canal (fissures, ulcers and stenoses), perianal fistulas and skin lesions (abscesses and skin tags) [13]. Knowing the anatomy of the fistulous path and its relationship with the other neighboring elements is critical for establishing the correct therapeutic behavior, thus preventing the recurrence of fistulas [14]. Pelvic MRI represents the most used imaging investigation for the characterization of perianal disease as a complementary method of examination under anesthesia (EUA) [15] that is considered the gold-standard diagnostic test in certain studies [14]. An essential role in the evaluation of perianal disease also belongs to EUS, with good sensitivity results in most studies [15], having the advantage that it also allows the assessment of the macroscopic aspect of the recto-colonic mucosa and the internal orifice of the fistulous tract [14]. However, its role is limited in the case of patients with stricturing phenotype [15]. At EUS, the classic appearance of fistulas corresponds to hypoechoic linear trajectories, sometimes with the description of hyperechoic foci at this level, determined by the presence of gas bubbles [14] or as a small hypoechoic round-shaped area in the intersphincteric space [8]; abscesses are described as anechoic or hypoechoic areas, which can sometimes present a hyperechoic capsule and echoic content determined by the existence of cellular debris [8]. In EUS, the best results are obtained with linear scanning probes, but the association of a radial examination can provide additional information [16]. Therefore, we suggest that EUS examination for perianal CD be performed, where possible, using both linear and radial scanning types. EUS presents difficulties in identifying suprasphincteric and posteriorly located fistulous trajectories [14], while for MRI, the limitations appear in evaluating short, superficial, anteriorly located or anovaginal trajectories [14,16,17].

However, the best results are obtained by combining at least two of the methods mentioned above (EUA, MRI and EUS) [15]; EUA associated with MRI or EUS is the most cost-effective and logical choice [16]. A landmark study that compared the effectiveness of the three methods (EUA, MRI and EUS) for the evaluation of perianal CD patients was that performed by Schwartz et al. in 2001 [16]. The study included 34 patients with known or suspected perianal CD, but a gold-standard consensus was established in only 32 patients. Diagnostic methods identified 40 fistulas and 13 abscesses for these patients. EUA and EUS correctly described the anatomy of fistulous tracts in 29 of 32 patients, while MRI did so in 26 of 30 patients. They concluded that there were no significant differences between the three methods, with the accuracy of each ranging between 87% and 91% (87% for MRI—confidence interval (CI), 69–96%; 91% for both EUS and EUA—CI, 75–98%), and when any two of the three methods are used in parallel, the accuracy approaches 100% [16].

A study by Orsoni et al. [18], which included 22 patients, compared the efficiency of EUS and MRI for identifying anorectal fistulas and abscesses that may complicate CD, showing a much more significant benefit for EUS. A total of 14 abscesses were identified by EUS and 9 by MRI, of which 11 were confirmed following surgical exploration. Regarding the number of fistulas, EUS identified 26, while MRI identified 14, and surgical exploration confirmed 27 fistulous tracts. Therefore, the study demonstrated a sensitivity of 100% for EUS and 55% for MRI to reveal the presence of abscesses, while for anorectal fistulas, the sensitivity was 89% for EUS and 48% for MRI. However, the study was published in 1999, and the current devices available for performing MRI have a much higher resolution, which is why we believe that the sensitivity of this scan for the evaluation of perianal CD is much higher than the results obtained in the previously presented study.

A more recent meta-analysis [19], which included four studies that compared EUS and MRI for the evaluation of perianal fistulas, demonstrated similar sensitivities for the two imaging studies (87% for both methods), but a higher specificity in the case of MRI for indicating the presence of fistulas. However, the results show low specificity values for establishing the diagnosis, both for EUS (43%) and MRI (69%). Similar efficiency between the two methods was also demonstrated by Molteni et al. [14]. Therefore, these results reinforce the idea of using a diagnostic imaging method complementary to surgical exploration for evaluating patients with perianal CD, which is necessary for the most accurate description of the anatomy of fistulous tracts [16].

Identifying fistulous tracts on EUS can be improved by using contrast agents, such as hydrogen peroxide. It can be injected during examination through the external cutaneous opening of the fistula, which generates bubbles with a hyperechoic aspect, thus facilitating the visualization of the fistula [20]. In a study carried out on a group of 21 patients, the use of hydrogen peroxide proved to be superior to the physical examination and standard ultrasonography, correctly describing the path of the fistula in the case of 20 patients; the results were later confirmed by surgery (95% accuracy) [21]. Similar results supporting the use of hydrogen peroxide for the presurgical evaluation of perianal fistulas have been approved by other studies [22,23,24].

Advances in ultrasonographic techniques have enabled the 3D reconstruction of the anal region during EUS. Zawadzki et al. [25] observed that, in some patients with perianal CD, the fistulas have a characteristic appearance on 3D EUS, appearing as a hypoechogenic tract encompassed by a hyperechogenic area with a thin hypoechogenic margin—“Crohn’s Ultrasound Fistula Sign” (CUFS). The study included 157 patients with perianal fistulas, of which 29 were also known to have CD. Among the patients with CD, CUFS was identified in 20 of them, while in the group of 128 patients without CD, CUFS was identified in only 3 cases. Therefore, this sign could distinguish different types of perianal fistulae; in the study mentioned above, CUFS had positive and negative predictive values for CD-associated fistulae of 87% and 93%, respectively [25]. Other signs, such as maximum width ≥4 mm of the fistulous tracts or the presence of a double tract or a fistulous tract bifurcation, have been described to differentiate CD fistulas from cryptoglandular fistulas [26].

The combination of 3D EUS and hydrogen peroxide could provide much more beneficial information. West et al. [27] compared the role of hydrogen-peroxide-enhanced three-dimensional endoanal ultrasonography and endoanal MRI for the evaluation of perianal fistulas, with the results provided by the two investigations being similar. There was a concordance between methods of 88% for the primary fistula tract, 90% for locating the internal opening of the fistula, 78% for secondary tracts and 88% for fluid collections [27]. Some authors suggest using Levovist as a contrast agent to evaluate anal fistulas, with promising results [28], or injecting aerated and diluted lidocaine gel [29]. Moreover, using the Doppler technique can provide additional data on the vascularization of perianal fistulas [24], information beneficial for surgical intervention.

EUS can also be used to monitor the healing of fistulas, thus guiding therapeutic decisions, such as when to stop medical treatment or when to remove the seton, with much more favorable results in terms of fistula closure, the risk of recurrence or the formation of perianal abscesses [30]. The most appropriate therapeutic approach for treating complex perianal fistulas associated with CD involves the combination of medical treatment, consisting of biological agents (e.g., infliximab), immunosuppressants and antibiotics, with surgical seton placement [31]. The use of infliximab without the association of surgical therapy may lead to the closure of the external fistula opening before the healing of the fistulous tract, which may favor the formation of abscesses and increase the rate of fistula recurrence [31].

In a study by Ardizzone et al. [32], 15 of the 30 patients with fistulizing CD included in the study presented closure of the external orifice of the fistula 10 weeks after the initiation of infliximab therapy (administered at weeks 0, 2 and 6). Still, only five showed closure of the fistulous tract at EUS. Furthermore, there was a higher rate of fistula recurrence in patients in whom the fistulous course persisted at this time interval [32]. Schwartz and colleagues [30] used EUS to monitor the evolution of fistulas during medical treatment associated with or without surgery, aiming to obtain a longer time interval without recurrence of fistulas and a lower incidence of the development of perianal abscesses in patients with CD. The treatment was stopped only when the EUS examination indicated the inactivity of the fistulas. The median time to EUS fistula healing was 21 weeks, with the authors suggesting that setons should be maintained for at least 5 months and patients should receive a minimum of five doses of infliximab to allow the fistulous tract to become inactive. Among the 21 patients in the study, 7 presented simple fistulas, which is why they only followed medical therapy. None showed signs of fistula recurrence at a median time of 47 weeks after discontinuation of infliximab. Thus, EUS could also be used to identify patients who associate a minimal risk of fistula recurrence with the discontinuation of infliximab therapy [30].

Similar conclusions were reached by Guidi et al. [31], with the average endosonographic fistula healing time being 28 ± 16 weeks (range 8–55 weeks) in the case of eight out of nine monitored patients. In the case of six patients, infliximab treatment was stopped after an average of 9.2 administered infusions, and five of them maintained the clinical and endosonographic response during an average follow-up period of 19.4 ± 8.8 months (range 3–28 months) since infliximab discontinuation. In 2008, Spradlin et al. [33] published a prospective randomized study performed on 10 patients with perianal CD, of which 5 patients were evaluated using EUS to guide the therapeutic attitude, and the rest of the patients were included in the control group and did not benefit from this investigation during monitoring. All patients initially underwent EUS to identify fistulous tracts. The medical treatment represented by azathioprine or 6-mercaptopurine, metronidazole (1500 mg a day) or ciprofloxacin (1000 mg a day) and infliximab were combined with surgical seton placement under EUS guidance for the experimental group or only by EUA (without knowing the result of EUS baseline examination) in the control group. The results showed that, after an average period of 99 days, four of five (80%) patients in the experimental group had complete cessation of drainage, with an average time to EUS evidence of fistula inactivity of 229 days, while in the control group, only one out of five (20%) patients had complete cessation of drainage [33]. The outcomes of these studies suggest that monitoring the healing of perianal fistulas based on clinical examination alone is associated with a higher risk for their recurrence or the development of other complications, and they recommend the use of EUS or other imaging methods, such as MRI, to guide combined medical and surgical therapy in order to obtain a lasting cure of perianal CD [20].

## 4. Differentiating Ulcerative Colitis and Crohn’s Disease

Starting from the idea that, in UC, the inflammatory process is limited to the mucosa and submucosa, while in CD, the inflammation is transmural and may affect all layers of the intestinal wall, EUS could be used for the differential diagnosis of the two entities in cases where a definitive diagnosis cannot be achieved based on the data provided by the clinical examination, laboratory tests, colonoscopy and histopathological examination.

Limberg [34] suggested that the main ultrasonographic criteria that can be used to differentiate between UC and CD are the maximum thickness of the intestinal wall and the parietal stratification. In CD, due to transmural inflammation, the thickening of the intestinal wall is more pronounced, along with the loss of the parietal layering. In UC, the thickening is not so marked in most cases, and the layers can be differentiated [34]. However, these characteristics do not apply to all IBD patients, who may have mild forms of CD with preservation of the parietal stratification, and severe forms of UC may be associated with the loss of stratification [34].

Clinical studies that evaluated the usefulness of EUS for the differentiation of IBD date back to the 1990s, when Hildebrandt et al. [35] examined 37 patients diagnosed with IBDU, aiming to differentiate mucosal from transmural inflammation. Mucosal inflammation was defined by the preservation of the parietal stratification and the thickening of the submucosa (this could be explained by the fact that the destruction of the mucosal epithelium or the presence of mucosal oedema allows the transmission of a more significant amount of ultrasound energy to the submucosa, which could lead to an overestimation of its thickness). In contrast, transmural inflammation was characterized by a loss of differentiation of the five ultrasonographic layers of the intestinal wall. Of the 37 patients included in the study, 14 underwent surgery, so the information obtained at EUS could be compared with that provided by the histopathological examination. It was found that the results obtained after the histopathological examination correlated with those supplied by EUS in all 14 cases, with 3 out of 14 patients presenting mucosal inflammation and the remaining 11 having transmural involvement.

Afterward, Gast and Belaïche [36] performed a prospective study to identify whether specific characteristics upon rectal EUS examination, such as bowel wall thickness, mucosa appearance, submucosa thickness, presence of blood vessels with increased diameter in the submucosa and presence of lymph nodes, could be used to differentiate the two main types of IBD. The intestinal wall thickening and alteration of the typical five-layer ultrasonographic structure of the bowel wall were identified in both CD and UC patients. In contrast to UC, there was a direct correlation between intestinal wall thickness and the Crohn’s Disease Activity Index (CDAI) for CD. Thus, a cutoff value of intestinal wall thickness of 5.5 mm was suggested to differentiate active disease (≥5 mm) from inactive disease (<5 mm). The main features identified that could be used to differentiate the two entities were the presence of a more significant number of blood vessels >2 mm in diameter in CD, which appears to persist even in patients in remission and the presence of a higher number of large lymph nodes in patients with UC. The presence of a single lymph node or the absence of lymph nodes was suggestive of CD, while identifying at least two lymph nodes was more specific for UC. In addition, the presence or absence of lymph nodes could differentiate the active form of UC (i.e., at least one lymph node) from the inactive form (i.e., no lymph nodes).

Regarding the use of lymph nodes for the differentiation of IBD, these results contradict those published in a more recent study by Ellrichmann et al. [37]. This study identified pathological lymph nodes in 14/19 patients (73.7%) with active CD but none in those with UC. A cause of these differences could be represented by the intestinal segment at which EUS was performed (mid-sigmoid colon for Ellrichmann et al. and rectum for Gast and Belaïche), considering that, classically, in CD, the rectum is spared from the inflammatory process. Other findings included thickening of the intestinal wall with the predominance of the mucosal layer in UC, respectively the submucosal layer in CD [37]. Combining a series of characteristics (thickness of the mucosa or submucosa, the total thickness of the intestinal wall and the presence of lymph nodes), the authors found a sensitivity of 0.93 (CI 0.78–0.98) and a specificity of 1 (CI 0.79–1) for the differentiation of active UC and active CD.

Similar results were also reported by Roushan et al. [38]. They described a sensitivity and specificity of mean mucosa thickness to distinguish between UC and CD of 92.3% and 88.6%, respectively, with a cutoff point of 1.1 mm (*p* < 0.001) and a sensitivity and specificity of mean submucosa thickness to distinguish between CD and UC of 100% and 86.1%, respectively, with a cutoff point of 1.08 mm (*p* < 0.001) [38]. Both studies reported positive statistical correlations between total bowel wall thickness and scores used to assess clinical and endoscopic activity [37,38].

Exciting data came from the study by Rustemovic and colleagues [39], who used transrectal endoscopic ultrasound (TRUS) elastography in an attempt to differentiate the two main types of IBD. They identified a significantly higher strain ratio in the rectal wall and adjacent tissue in patients with active CD compared to the group formed by patients with active UC. The authors also described a significant difference in rectal wall thickness between CD patients without rectal involvement and the control group, and this could suggest a potential predictive role of TRUS in identifying CD patients who may develop rectal involvement during the course of the disease or perianal disease.

Although the results of the studies mentioned above are promising, currently, there are no firm EUS criteria to differentiate UC from CD.

## 5. Monitoring the Activity of Inflammatory Bowel Disease

The assessment of IBD activity is mainly based on entity-specific clinical or endoscopic severity scores. Still, ultrasonography can be of real benefit, especially if an evaluation of transmural healing is sought.

As mentioned in one previously reviewed article [36], intestinal wall thickness could be used to differentiate active from inactive disease. This was observed when Rasmussen and Riis [40] found an increase in rectal wall thickness directly proportional to clinical, endoscopic and histological severity in UC patients.

In an attempt to establish the role of TRUS in assessing the severity of UC, Dağli et al. [41] proposed a series of cutoff values of the total thickness of the intestinal wall and the thickness of the mucosa and the submucosa to allow for the differentiation of patients in remission from those with active disease. The cutoff values for active UC were ≥5.36 mm for total wall thickness, ≥2.23 mm for mucosa thickness and ≥2.34 mm for submucosa thickness. The presence of arterial or venous flow at the level of the submucosa, highlighted using Doppler ultrasonography, is another valuable parameter for the differential diagnosis, which, together with the thickening of the submucosa, showed the highest specificity and sensitivity compared to the other evaluated parameters for differentiating active UC from UC in remission.

Soweid et al. [42] evaluated IBD activity by using catheter-probe-assisted endoluminal ultrasonography. While examining 7 UC patients and 11 CD patients, they found a strong correlation between bowel wall thickness and colonoscopic appearance of the mucosa (r = 0.84, *p* = 0.02) and a moderate correlation between bowel wall thickness and clinical activity scores (r = 0.65, *p* = 0.11) in the case of UC; they also found a strong correlation between parietal stratification loss and clinical activity scores (r = –0.80, *p* = 0.003) and a moderate correlation between intestinal wall thickness and histological changes (r = 0.62, *p* = 0.04) in the case of CD. Ultrasonographic changes on EUS mainly involved the first three layers of the intestinal wall, corresponding to the mucosa and submucosa, in UC patients. At the same time, a unique feature was identified for CD, namely marked thickening of the fourth layer that corresponds to the muscularis propria [42].

Similar to the previous study, Rana et al. [43] reported a significant correlation between the total wall thickness, mucosa + submucosa thickness, and clinical and endoscopic severity scores in UC patients. Regarding bowel wall stratification, there was only a correlation between loss of regular stratification and severity of endoscopic appearance, but not for clinical activity scores.

Cho et al. [44,45] observed that, in the case of patients with active UC, the intestinal wall can associate hypoechoic changes with EUS, predominantly affecting the mucosa and submucosa. Related to the thickening of the intestinal wall, these hypoechoic changes tend to advance to the deeper layers, concomitantly with the severity of UC. Based on these changes, they classified active UC as follows: UC-M—thickened intestinal wall with intact parietal layer (inflammation is limited to the mucosa); UC-SM—thickened intestinal wall with hypoechoic changes extended to the superficial part of the third layer (inflammation affects the submucosa); UC-SMdeep—thickened intestinal wall with hypoechoic changes extended more profound in the third layer compared to the previous stage (inflammation affects the deep submucosa); UC-MP—thickened intestinal wall with hypoechoic changes extended up to the level of the fourth layer (inflammation affects the muscularis propria); and UC-SS/SE—thickened intestinal wall with hypoechoic changes exceeding the fourth layer (inflammation penetrates the muscularis propria). The authors suggest that patients with changes suggestive of UC-SMdeep, UC-MP or UC-SS/SE require careful monitoring, as most patients who required surgery were in these stages. However, this statement may no longer be applicable, considering the progress of medical therapies in recent years.

Moreover, for UC, Tsuga et al. [10] proposed a classification based on the aspect of the delimitation of the layers of the intestinal wall (mucosa, submucosa and muscularis propria), describing the transition from one layer to another as “smooth”, “irregular” or “blurred”. According to these changes, 6 degrees of severity have been identified. However, there was only a modest correlation between the score proposed by Tsuga and the Matts endoscopic score [46] or the clinical severity scores, as also obtained by a later study by Hurlstone et al. [47].

Starting from the classification proposed by Tsuga, Yan and colleagues [48] developed an EUS score for the assessment of UC severity, which evaluates the following parameters: the thickness of the intestinal wall, the depth of the inflammatory process and hyperemia defined by the vascularization of the intestinal wall during the power Doppler examination (Table 1).

Recent studies have demonstrated a significant positive correlation between this EUS score and clinical (Truelove and Witts, Mayo) and endoscopic (UC endoscopic index of severity—UCEIS) severity scores, suggesting that it can also be used to monitor treatment response [49]. In this sense, Higaki et al. [50] demonstrated that, in patients with UC in remission, a greater thickness of the mucosa and submucosa upon EUS examination is associated with a higher risk of relapse (the mean rectal wall thickness was 2.73 mm (2.13–3.33 mm) in patients who presented a relapse). On the other hand, Watanabe et al. [51] observed that, in a group of patients with severe UC refractory to corticosteroids and treated with cyclosporin A, those who showed greater rectal mucosa thickness on EUS had a better response to therapy. Other authors suggest that EUS could help predict the response of patients with active UC to medical treatment and to identify patients who will require surgical intervention (mainly cases in which EUS reveals an inflammatory process involving the muscularis propria or deeper layers) [52]. However, further studies are needed to reach a consensus regarding these results.

## 6. EUS and Inflammatory-Bowel-Disease-Related Complications

It is well-known that one of the long-term complications associated with IBD is the development of colorectal cancer. The first case in the literature that described the role of EUS for the evaluation of IBD-associated tumors dates back to the late 1990s, when Shimizu et al. [53] were able to diagnose, using EUS, an invasive rectal carcinoma in a patient with a history of 19 years of extensive UC throughout the colon. Recently, Kobayashi [54] conducted a study on a group of 13 patients with UC that associated 16 colorectal tumors to identify the usefulness of EUS in evaluating these lesions. EUS correctly described the depth of invasion in 15 out of 16 lesions (94% accuracy), suggesting that EUS could be used to select the patients who may benefit from less invasive therapeutic methods, such as endoscopic resection, considering that surgery may associate complications that can severely affect the quality of life.

Other complications that may occur in the evolution of patients with IBD are represented by the development of abscesses or strictures/stenoses, more frequently in the case of CD. EUS is widely used with success to drain collections that may complicate pancreatic disease. Due to the transmural inflammatory process in CD and the risk of fistula formation, EUS-guided drainage of abdominal abscesses has not been routinely used for this category of patients [55]. However, several cases are described in the literature wherein the EUS-guided drainage of abdominal fluid collections in CD, using plastic stents or lumen-apposing metal stents (LAMS), was successfully performed [55,56,57,58,59]. In addition, Monino et al. [60] performed, with excellent results, an EUS-guided gastrojejunal anastomosis, using LAMS for duodenal stricture, in a patient with refractory CD. All of these cases emphasize the benefits of EUS for patients with IBD, so in addition to the diagnostic role, this investigation can also be used for an intervention in this category of patients.

## 7. Limitations of EUS in Inflammatory Bowel Disease

EUS is a technique that provides beneficial and complementary information to other imaging investigations used to evaluate pathologies of the digestive system. Still, it represents a method that requires a complex learning curve that demands years of high-level training [61]. Therefore, the main limitation of EUS is that it is an operator-dependent method, and experience in evaluating IBD is limited.

Because the two standard types of echoendoscopes, linear and radial, are side-viewing instruments, the evaluation of the lower digestive tract employing EUS is usually limited to the level of the rectum [7]. Thus, EUS finds better utility in monitoring UC, where the inflammatory process affects the rectum and can extend to the left colon, and less often to the right colon. However, this barrier can be overcome by using forward-viewing linear echoendoscope (FV-EUS) or EUS mini probes [7]. FV-EUS presents an angulation range of up to 180°, and the hard tip of the echoendoscope is shorter, thus allowing easier handling and evaluation of areas difficult to reach with conventional echoendoscopes, even of the entire colon [62,63]. In addition, the forward orientation of the endoscopic and ultrasonographic view could improve the interventional procedures, with the perpendicular axis easing the advancement of accessories through the working channel [62]. Still, the absence of an elevator and the reduction of the ultrasonographic visual field from 180° to 90° could represent limitations of FV-EUS [62,63]. EUS mini probes can be used during standard colonoscopy, being inserted through the colonoscope’s instrument channel [7]. Due to their small caliber, they can be of utmost importance in the case of stricturing CD. The mini probes’ ultrasound frequency can reach up to 20 MHz [9], obtaining a higher resolution that offers a superior characterization of superficial lesions but associates the disadvantage of a lower penetrability of ultrasounds in depth [9]. In addition, they do not allow for fine-needle aspiration or biopsy to be performed [64], and they present a limited number of uses, with a viability of 50–100 procedures [65]. Therefore, some authors suggest that if conventional echoendoscopes are available and the disease phenotype permits their use, EUS mini probes should not replace them [64].

On the other hand, another limitation of EUS for this category of patients is identified in the use of the method to differentiate the two entities of IBD, especially in severe forms of the disease, when, in both CD and UC, the thickening of the intestinal wall may be associated with the loss of typical stratification. Therefore, based only on the evaluation of the two previously mentioned parameters, the usefulness of EUS for the differentiation of severe forms of IBD is reduced, which is why further studies should identify other features that could be used for this purpose.

## 8. Conclusions

Currently, the primary use of EUS in patients with IBD is represented by the characterization of perianal CD. However, all the studies discussed previously support a great potential of the applicability of EUS for this group of patients (Table 2). Therefore, studies should focus more frequently on this direction, aiming to perfect the role of EUS in diagnosing and treating IBD patients.

## Figures and Tables

**Table 1:** Yan et al.’s [48] revised EUS score for ulcerative colitis.

*Component*	*Score*
***Bowel wall thickening***	
*Normal* ≤3.0 mm	0
*Mild thickening* 3.1–4.0 mm	1
*Moderate thickening* 4.1–6.0 mm	2
*Severe thickening* >6.0 mm	3
***Depth of inflammation***	
*Superficial:* No disruption of the 5-layer echo pattern	0
*Subepithelial:* Disruption of the first 3 layers to the submucosa but not beyond	1
*Deep:* Disruption beyond the submucosa to the muscularis propria	2
*Transmural:* Disruption beyond the muscularis propria to the serosa or beyond	3
***Hyperemia***	
*Normal:* Absence of intramural vascular signal	0
*Mild:* Intermittent signal	1
*Moderate:* Continuous signal	2
*Severe*: Presence of intramural anechoic vessel seen without power Doppler, with immediate continuous signal on power Doppler	3
*TOTAL*	9

**Table 2:** Possible applications of EUS in inflammatory bowel disease.

**Perianal Crohn’s disease** ◦ EUA associated with MRI or EUS provides the best results about the anatomy of the fistulous path and its relationship with the other neighboring elements; ◦ The combination of 3D EUS and contrast agents (e.g., hydrogen peroxide) could provide beneficial information; ◦ EUS can be used to monitor the healing of fistulas, guiding therapeutic decisions, such as when to stop medical treatment or when to remove the seton. **Differentiating ulcerative colitis and Crohn’s disease** ◦ Currently, there are no firm EUS criteria to differentiate UC from CD; ◦ Bowel wall thickness, mucosa appearance, submucosa thickness, presence of blood vessels with increased diameter in the submucosa and presence of lymph nodes could be used to differentiate the 2 main types of IBD; ◦ Transrectal endoscopic ultrasound elastography may be helpful in the differential diagnosis. **Monitoring the activity of inflammatory bowel diseases** ◦ Yan et al. [48] developed an endoscopic score for the assessment of UC severity, which evaluates the thickness of the intestinal wall, the depth of the inflammatory process and hyperemia defined by the vascularization of the intestinal wall during the power Doppler examination. **EUS and inflammatory bowel disease-related complications** ◦ EUS may help diagnose IBD-associated tumors; ◦ Abdominal fluid collections developed as complications of CD could be treated by EUS-guided drainage using plastic stents or lumen-apposing metal stents (LAMSs); ◦ Monino et al. [60] performed an EUS-guided gastrojejunal anastomosis using LAMS for duodenal stricture in a patient with refractory CD.

## Data Availability

Not applicable.
